# Editorial: Multisensory Integration: Brain, Body, and World

**DOI:** 10.3389/fpsyg.2015.02046

**Published:** 2016-01-12

**Authors:** Achille Pasqualotto, Magda L. Dumitru, Andriy Myachykov

**Affiliations:** ^1^Faculty of Arts and Social Sciences, Sabanci UniversityIstanbul, Turkey; ^2^Department of Cognitive Science, Macquarie UniversitySydney, NSW, Australia; ^3^Cognitive Science Department, Graduate School of Informatics, Middle East Technical UniversityAnkara, Turkey; ^4^Department of Psychology, Northumbria University NewcastleNewcastle-upon-Type, UK; ^5^School of Psychology, Centre for Cognition and Decision Making, National Research University Higher School of EconomicsMoscow, Russia

**Keywords:** multisensory integration, body representation, attentional deployment, emotional processing, numerical cognition, language, embodied reasoning, time processing

The brain is safely sealed inside the cranium, with virtually no direct interaction with other parts of the body and the outside world. Nevertheless, it constantly processes the information conveyed by several sensory modalities in order to create representations of both body and outer world and to generate appropriates motor responses (Ehrsson et al., 2005; Farnè et al., 2005; Green and Angelaki, 2010). For example, vision can convey information about dangerous stimuli to trigger the generation of appropriate motor response (e.g., escape, avoidance, fight, etc.). Rather than processing sensory inputs in isolation, the brain *integrates* sensory information (Stein and Meredith, 1993; Fetsch et al., 2012) by forming reliable and robust representation of the external world and body. For example, when both visual and auditory input inform about the same danger, an appropriate motor response is more rapid and efficient (Sereno and Huang, 2006; Laing et al.).

Until a few decades ago, it was strongly believed that sensory (or multisensory) integration occurred only in high-level/associative areas or the cortex (Ghazanfar and Schroeder, 2006; Pavani and Galfano). Recently, several “new” multisensory areas have been discovered (Gobbelé et al., 2003; Pietrini et al., 2004), suggesting that a larger portion of the cortex is engaged in multisensory processing. Additional evidence suggests that multisensory integration also occurs in sub-cortical areas (Kuraoka and Nakamura, 2007; Amad et al., 2014). Finally, and perhaps surprisingly, some studies have demonstrated that multisensory processing occurs in primary sensory areas that were traditionally considered to be *uni-sensory* (Zangaladze et al., 1999; Murray et al., 2005).

Theories such as “neural reuse” (Anderson, 2010) and “metamodal” organization of the brain (Pascual-Leone and Hamilton, 2001) attempt to provide new paradigms for brain functioning taking into account widespread multisensory integration. The evolutionary advantage of multisensory integration might be the resulting availability of more reliable representations of the external world and body (Elliott et al., 2010; Grüneberg et al.) based on multiple sensory inputs and the resilience to brain injuries and sensory loss (Sarno et al., 2003; Pasqualotto and Proulx, 2012; Brown et al.; Finocchietti et al.). Indeed, multisensory integration has been reported in various experimental tasks including spatial representation (Pasqualotto et al., 2005), object recognition (Woods and Newell, 2004; Harris et al.; Höchenberger et al.; Laing et al.), movement perception (Grüneberg et al.; Imaizumi et al.; Uesaki and Ashida), body representation (Pasqualotto and Proulx, 2015; Pavani and Galfano; Tajadura-Jiménez et al.; Yiltiz and Chen), emotional processing (Miu et al.; Piwek et al.), attentional deployment (Spence, 2002; Depowski et al.), language (Gallese, 2008; Myachykov and Tomlin, 2008; Myachykov et al., 2012; Lam et al.; Shaw and Bortfeld), embodied reasoning (Dumitru, 2014), sensory awareness (Cox and Hong), numerical cognition (Dumitru and Joergensen), auditory perception (Brogaard and Gatzia), and time perception (Homma and Ashida).

The articles included in this special issue offer novel insights about recent developments within the field of multisensory integration, and we believe that they will help understanding the multisensory nature of brain functioning.

## Author contributions

AP wrote the first draft of the manuscript. MD and AM provided comments, additions, and further improvements. All authors have approved the final version of the manuscript.

### Conflict of interest statement

The authors declare that the research was conducted in the absence of any commercial or financial relationships that could be construed as a potential conflict of interest.
